# An international, stepped wedge, cluster-randomized trial investigating the 0/1-h algorithm in suspected acute coronary syndrome in Asia: the rational of the DROP-Asian ACS study

**DOI:** 10.1186/s13063-022-06907-4

**Published:** 2022-12-07

**Authors:** Kenji Inoue, Jack Tan Wei Chieh, Lim Chiw Yeh, Shuo-Ju Chiang, Arintaya Phrommintikul, Pannipa Suwanasom, Sazzli Kasim, Bakhtiar Ahmad, Alzamani Mohammad Idrose, Farina Mohd Salleh, Shunsuke Oyamada, Yohei Hirano, Shohei Ouchi, Moriyuki Terakura, Naoyuki Yokoyama, Ken Kozuma, Mamoru Nanasato, Ryosuke Higuchi, Kazuhiko Yumoto, Tomoyuki Fukuzawa, Issei Shimada, Evangelos Giannitsis, Raphael Twerenbold, Tohru Minamino

**Affiliations:** 1 Department of Cardiovascular Biology and Medicine, Juntendo University Nerima Hospital, Tokyo, Japan; 2 Department of Cardiology, National Heart Centre Singapore and Sengkang General Hospital, Singapore, Singapore; 3 Division of Cardiology, Department of Internal Medicine, Taipei City Hospital Yangming Branch, Taipei, Taiwan; 4 Division of Cardiology, Department of Internal Medicine, Faculty of Medicine, Chiang Mai University, Chiangmai, Thailand; 5 Division of Cardiology, Hospital Al-Sultan Abdullah, University Teknologi MARA, Kuala Lumpur, Malaysia; 6 Division of Emergency, Kuala Lumpur Hospital, Kuala Lumpur, Malaysia; 7 Division of Emergency, Institut Jantung Negara, Kuala Lumpur, Malaysia; 8 Departments of Biostatistics, JORTC Data Center, Tokyo, Japan; 9 Department of Emergency and Critical Care Medicine, Juntendo University Urayasu Hospital, Chiba, Japan; 10 Department of Cardiovascular Biology and Medicine, Juntendo Urayasu Hospital, Chiba, Japan; 11 Department of Emergency, Teikyo University School of Medicine, Tokyo, Japan; 12 Department of Cardiology, Teikyo University School of Medicine, Tokyo, Japan; 13 Department of Cardiology, Sakakibara Heart Institute, Tokyo, Japan; 14 Department of Cardiology, Yokohama Rosai Hospital, Kanagawa, Japan; 15 Shimada General Hospital, Chiba, Japan; 16 Department of Cardiology, University Hospital Heidelberg, Heidelberg, Germany; 17 Department of Cardiology and University Center of Cardiovascular Science, University Heart and Vascular Center Hamburg, Hamburg, Germany; 18 Department of Cardiovascular Biology and Medicine, Juntendo University Graduate School of Medicine, Tokyo, Japan

**Keywords:** 0-h/1-h algorithm, High-sensitivity troponin T, Non-ST elevation of acute coronary syndrome, Overcrowding emergency department

## Abstract

**Background:**

More than half of the world’s population lives in Asia. With current life expectancies in Asian countries, the burden of cardiovascular disease is increasing exponentially. Overcrowding in the emergency departments (ED) has become a public health problem. Since 2015, the European Society of Cardiology recommends the use of a 0/1-h algorithm based on high-sensitivity cardiac troponin (hs-cTn) for rapid triage of patients with suspected non-ST elevation acute coronary syndrome (NSTE-ACS). However, these algorithms are currently not recommended by Asian guidelines due to the lack of suitable data.

**Methods:**

The DROP-Asian ACS is a prospective, stepped wedge, cluster-randomized trial enrolling 4260 participants presenting with chest pain to the ED of 12 acute care hospitals in five Asian countries (UMIN; 000042461). Consecutive patients presenting with suspected acute coronary syndrome between July 2021 and Apr 2024 were included. Initially, all clusters will apply “usual care” according to local standard operating procedures including hs-cTnT but not the 0/1-h algorithm. The primary outcome is the incidence of major adverse cardiac events (MACE), the composite of all-cause death, myocardial infarction, unstable angina, or unplanned revascularization within 30 days. The difference in MACE (with one-sided 95% CI) was estimated to evaluate non-inferiority. The non-inferiority margin was prespecified at 1.5%. Secondary efficacy outcomes include costs for healthcare resources and duration of stay in ED.

**Conclusions:**

This study provides important evidence concerning the safety and efficacy of the 0/1-h algorithm in Asian countries and may help to reduce congestion of the ED as well as medical costs.

## Introduction

Emergency department (ED) overcrowding is a major public health problem in Asian countries and has been associated with poor outcomes, increased resource use, and restricted access to care [1–3]. Chest pain is one of the most frequent reasons for emergency department (ED) visits. These patients are frequently managed in the ED to rule out an acute coronary syndrome (ACS). However, ACS is diagnosed only in a minority (about 5–20%, depending on the clinical setting) while the vast majority does not suffer from a life-threatening cause of chest pain [4]. As a consequence, a large number of patients are managed in the hospital inappropriately [5]. This practice produces an increase in costs and overcrowds the ED, with a negative impact on the patients and the healthcare system. Waiting times are a pressing societal problem and efficient treatment pathways, which are essential to ensure the accurate, timely, and cost-effective early management of ACS patients. Conversely, missed diagnosis and treatment inefficiency are associated with increase morbidity, mortality, and costs [6, 7].

Based on the above, efficient patient stratification approaches are needed. The population composition and economic environment varies widely in a given population, so such stratification approaches should be kept as simple as possible. The European Society of Cardiology (ESC) guidelines recommend the use of high-sensitivity cardiac troponin (hs-cTn) 0-h/1-h algorithms in patients presenting with suspected non-ST elevation ACS (NSTE-ACS) as Class I, Level B since 2015 [8, 9]. This algorithm was based on conceptual considerations given the ongoing clinical implementation of hs-cTn assays at that time and was initially supported only by limited data [10]. Due to the lack of data from Asian countries, which differ substantially from Central European countries regarding patient’s characteristics and healthcare systems, the 0/1-h algorithm is not recommended by Asian guidelines [11–13]. Accordingly, most patients presenting with suspected ACS in Asian countries are still assessed with serial retesting of hs-cTn after 2, 3, or even 6 h, which is not only more time consuming, but has been described to be less safe in comparison to the ESC 0/1-h algorithm [14].

The “*d*iagnosis and *r*eduction of *o*vercrowding emergency de*p*artment in *Asia*n *a*cute *c*oronary *s*yndrome based on the 0-1 algorithm using high-sensitivity troponin T assay” (DROP-Asian ACS) study is a stepped wedge, cluster-randomized trial, which is currently being conducted to compare the real-world safety and efficacy of the usual care with the 0/1-h algorithm in patients with suspected non-ST elevation ACS (NSTE-ACS) (UMIN; 000042461). The findings will help to clarify how to safely reduce ED dwelling time and medical costs in order to achieve the most efficient level of care for patients in the ED.

## Methods

### Follow-up and clinical end endpoints

After hospital discharge, patients were contacted after 30 days by telephone calls or appointments.

#### Primary outcome measure

The primary aim of the present study is to determine the relative safety of the 0/1-h algorithm compared with the current standard of care for patients presenting with suspected NSTE-ACS to the ED. Primary outcome measure is the incidence of major adverse cardiac events (MACE), defined as the composite of cardiovascular death, new acute myocardial infarction (AMI) (type 1 or 2), unstable angina, or unexpected revascularization within 30 days (defined as any unplanned hospitalization with cardiac revascularization performed within the first 12 h after hospital admission in the context of ACS). Of note, AMI diagnosed within 12 h of index presentation among participants continuously in-hospital will be considered as the index presenting AMI and not included as an endpoint event. An AMI documented to have commenced outside this time (recurrent/new AMI type 1 or 2) in hospitalized patients or within 12 h of index presentation among participants already discharged from the hospital (missed AMI type 1 or 2) will be considered an endpoint event.

#### Secondary outcome measures

The following predefined secondary outcomes are considered:

1. The proportion of patients managed as outpatients in order to quantify the efficacy of the investigated approaches in both arms.
2. The time from ED presentation to discharge in outpatients. The ED dwell time is derived from a common electronic patient record system used across all participating sites and is calculated as the time of the medical examination between the start time (sign-in time: defined as first medical contact with a MD or the triage nurse) and the end time (sign-off time: defined as the time when the attending physician has finished all medical procedures in the ED, including charting, with the patient to ready to leave the examination room either to be discharged or transferred to a different unit/ward within the hospital).
3. All-cause mortality (cardiovascular death and non-cardiovascular death) and new myocardial infarction (type 1 or 2) within 30 days after the index presentation.
4. Incidence of any cardiac revascularization within 30 days after the index presentation.
5. Incidence of ED representation or hospitalization for unstable angina within 30 days after the index presentation.
6. Costs for healthcare resource use within 30 days following the index ED presentation are calculated based on each country’s guidelines and cost tables for hospitals. Different costs are used for academic and general hospitals. For each patient, the costs are calculated based on the observed number and type of healthcare resources used.
7. Incidence of cardiac examinations (e.g., stress testing or coronary angiography) within 30 days after the index presentation. Adherence to the intervention condition (the 0/1-h algorithm) was evaluated.

### Study design and population (Fig. 1)

This is a prospective, international randomized trial involving eleven hospitals (*Japan*; Juntendo University Nerima Hospital, Juntendo University Urayasu Hospital, Sakakibara Heart Institute, Shimada general hospital, Teikyo University School of Medicine, Yokohama Rosai Hospital, *Singapore*; National Heart Centre Singapore and Sengkang General Hospital, *Thailand*; Maharaj Nakorn Chiangmai Hospital, *Taiwan*; Taipei City Hospital Yangming Branch, *Malaysia*; University Teknologi MARA Teaching Hospital, Kuala Lumpur Hospital, and Institute Jantung Negera) from five Asian countries. These hospitals provide advanced interventional therapy to patients presenting with suspected ACS. Patients of ≥18 years of age who presented to the ED of participating hospitals with symptoms suggestive of NSTE-ACS (e.g., chest pain, epigastric pain, radiating pain to the neck or shoulder, shortness of breath) are eligible for enrollment in the DROP-Asian ACS study, if the onset of symptoms occurred within the last 12 h. Patients were excluded from the present study if they presented with ST elevation myocardial infarction, cardiogenic shock, on chronic hemodialysis, or chest pain following chest trauma, or if they were unwilling or unable to provide their informed consent. All participating patients or their legally authorized representatives will provide written informed consent. This study is conducted according to the principles of the Declaration of Helsinki. Ethical approval has been received from the local ethics committees. This study is registered in the Japanese Trial Registry (Reg. No. UMIN ID; UMIN000042461; https://upload.umin.ac.jp/cgi-openbin/ctr_e/ctr_view.cgi?recptno=R000048466).

**Fig. 1:**
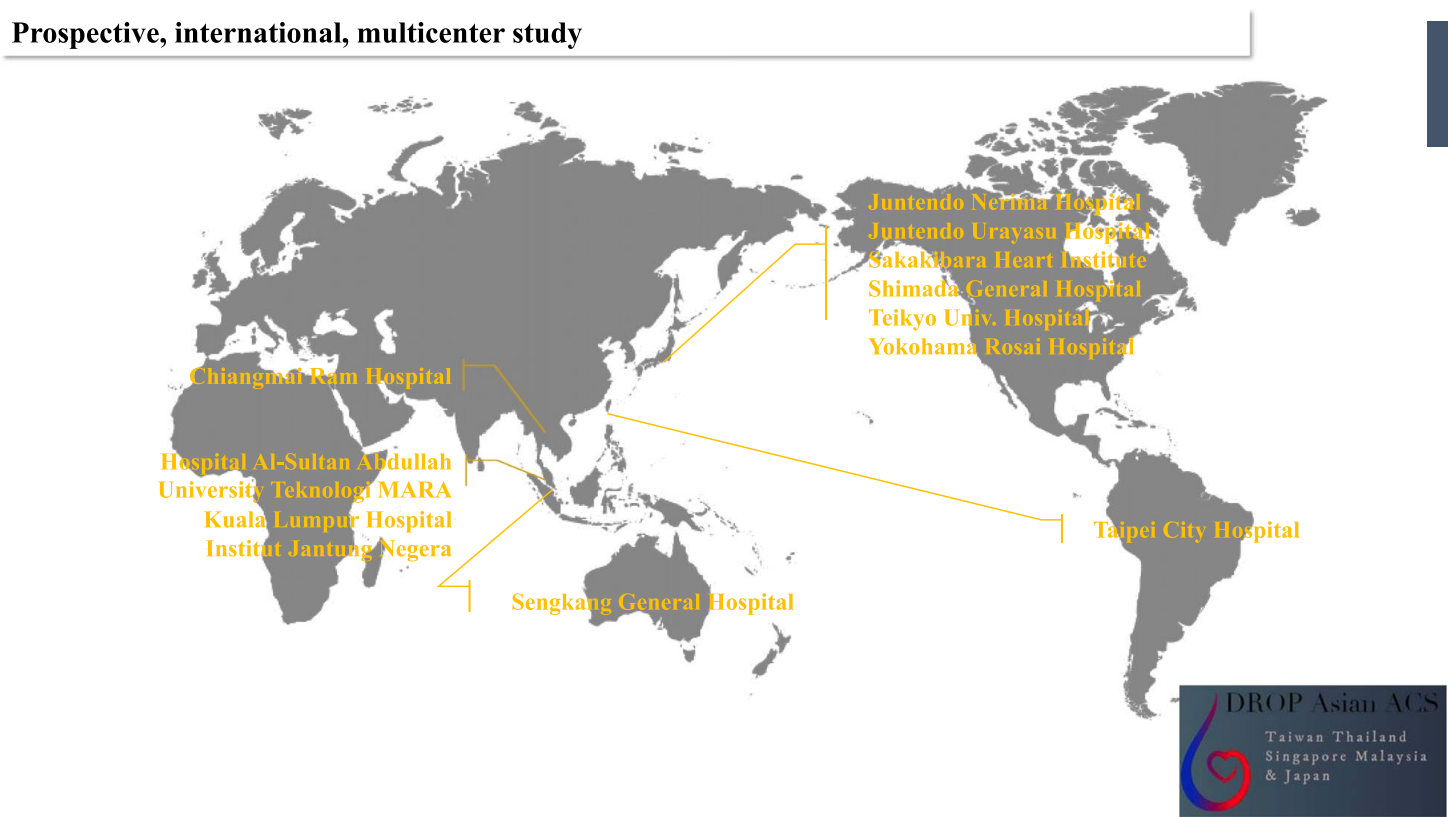
Participants. Six hospitals from Japan, and 3 hospitals from Malaysia join. Juntendo Urayasu Hospital and Shimada general hospital is in one cluster due to highly comparable geographical conditions (Chiba, Japan) and the expected relatively low number of enrollments (They can enroll patients during Monday to Friday, 9 a.m. to 4.p.m.)

### Routine clinical assessment

All study patients will undergo initial clinical assessments along with completion of the case record form, which includes a physical examination, documentation of symptoms, clinical history, cardiovascular risk factors, 12-lead electrocardiography (ECG), bedside cardiac ultrasound sonography (visual ejection fraction and an evaluation of the wall motion), chest X-ray, blood pressure, heart rate, and standard blood tests. Hs-cTnT (manufactured by Roche Diagnostics, Rotkreuz, Switzerland) is routinely determined and results provided to the treating physicians as part of the local standard of care in all participating centers in both treatment groups. This hs-cTnT-assay has a 99th percentile concentration of 14 ng/L with a corresponding coefficient of variation of 10% at 13 ng/L [12]. Limit of blank and limit of detection have been determined to be 3 and 5 ng/L. The physicians will complete the modified History, Electrocardiogram, Age, Risk factors and Troponin (HEART) score and the ED Assessment of Chest pain Score (EDACS) [15–17], and the HEART score will be further modified using the hs-cTnT level [18].

### Stepped wedge design

**Fig. 2:**
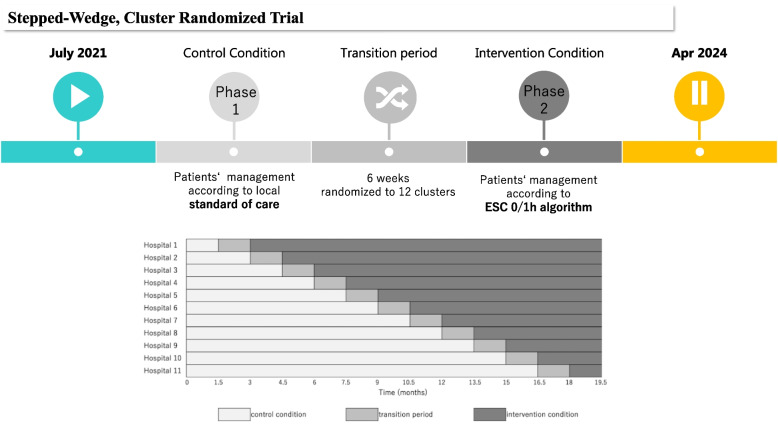
Study design. This study consists of 11 clusters with 12 hospitals. At study start, all clusters will apply “usual care” (control condition) to all patients, i.e., risk assessment and subsequent management includes hs-cTnT but not the application of the 0/1-h algorithm. One randomly allocated cluster will sequentially start to follow the 0/1-h algorithm after a transition period of 1.5 months in all chest pain patients (intervention condition; 0/1 care)

This study has a stepped wedge design, which is a type of cluster-randomized trial. This design gives all participating general practitioners the opportunity to use the intervention during the study. The group of hospitals to which the intervention applies will be called a “cluster.” Basically, each cluster contains one hospital, except for one cluster containing two hospitals—Shimada General Hospital and Juntendo University Urayasu Hospital—which are included in one cluster due to highly comparable geographical conditions (Chiba, Japan) and the expected relatively low number of enrollments because they can enroll the patients from Monday to Friday (9 a.m. to 4 p.m.) in both centers. Therefore, this study consists of 11 clusters with 12 hospitals. At study start, all clusters will apply “usual care” (control condition) to all patients, i.e., risk assessment and subsequent management includes hs-cTnT but not the application of the 0/1-h algorithm. Every 1.5 months, one randomly allocated cluster will sequentially start to follow the 0/1-h algorithm after a transition period of 1.5 months in all chest pain patients (intervention condition; 0/1 care) (Fig. 2).

### Sample size, level of significance, and power

A survey of ED physicians found the tolerable rate of missed major adverse cardiac events to be less than 1% [19]; however, an analysis by Kline et al. showed that a 2% rate of missed diagnoses of ACS might be considered acceptable considering the risk of harm from further testing [20], and more than 2% of ACS patients are inappropriately discarded annually according to a Medicare database [21]. Regional differences in the management of NSTE-ACS persist in Asian countries even after extensive adjustment for baseline characteristics and treatment [12]. Indeed, in previous studies, the MACE rates tend to be higher than in Central European or North American countries and vary from 1 to 4% [10, 18, 22, 23]. We estimated that, in the standard care arm, patients managed as outpatients by usual care would experience an event rate of 3%, for the primary outcome of MACE including all-cause death, subsequent myocardial infarction, unstable angina, or unexpected revascularization in 30 days. The non-inferiority margin of 1.5% is based on clinical judgment and the available literature, thus, accepting an upper limit of the 95% confidence interval. No assumption was made about the level of intra-cluster correlation coefficient (ICC) [24]. Based on these numbers, a one-sided significance level of 5% and a power of 80%, the required total sample size for a stepped wedge design with 11 clusters was determined to be 4260.

### Recalculation of sample size, level of significance, and power due to outbreak of severe acute respiratory syndrome coronavirus 2(SARS-CoV2)

Due to the influence of SARS-CoV2, the recruitment of Chiba, Chiang-Mai hospital, and Taipei city hospital has been sluggish. When the sample size was redesigned about 2 months after the start of the test, the power dropped to 67%. The SARS-CoV2 situation is unpredictable and we considered the possibility of reduced recruitment in other clusters. As a result of discussions within the research group, a non-randomized facility was added as the 11th cluster (Institute Jantung Negara) to ensure power. Furthermore, we extended each duration of the step from 1.5 to 2.5 months from the 12th steps. The overall “almost” randomized study is a limitation, but as a result, the power increased to 80%.

### Control condition: usual care

Based on current Asian guidelines, patients in the control condition will undergo “standard care” including hs-cTnT testing at time of ED presentation, which will be repeated 2–3 h after presentation (with further testing at the clinician’s discretion) according to respective local standard operating procedures. Patients will have their disposition determined by the treating ED clinician. Access to outpatient care will be as per local practice. Subsequent care will be physician-determined (Table 1). We are opting out of reviewing the patient data. Subjects have the right to withdraw from the study at any time for any reason.

**Table 1:** Physician-determined care

Hospital name	Country	Biomarker	Serial measurements	Risk stratification tool and definition of low-risk	Thereshold to rule in and role out MI
Juntendo University Nerima Hospital	Japan	Roche Elecsys hs-cTnT	None	HEART score ≤ 3, depends on an attending physicians	Less than 14 ng/L
Juntendo University Urayasu Hospital	Japan	AQT90 FLEX cTnT	Depends on an attending physicians (within 6 h)	HEART score ≤ 3 or TIMI< 2; depends on an attending physicians	Less than 99th percentile
Sakakibara Heart Institute	Japan	Roche Elecsys hs-cTnT	None	None	Less than 14 ng/L
Shimada General Hospital	Japan	Roche TROP T Sensitive test	None	None	Negative
Teikyo University Hospital	Japan	Roche Elecsys hs-cTnT	3 h	None	Less than 14 ng/L
Yokohama Rosai Hospital	Japan	Roche Elecsys hs-cTnT	3 h	None	Less than 14 ng/L
NHCS and Sengkang General Hospital	Singapore	Roche Elecsys hs-cTnT	2 h	EDACS < 16; Delta Troponin rise < 5 point (depends on the attending physician)	Male ≤29 ng/L, Female ≤19 ng/L
Maharaj Nakorn Chiangmai Hospital	Thailand	Roche Elecsys hs-cTnT	Depends on an attending physicians (within 6 h)	None	Less than 14 ng/L
Taipei City Hospital Yangming Branch	Taiwan	Roche Elecsys hs-cTnT	Depends on an attending physicians (within 6 h)	None	Less than 14 ng/L
UiTM	Malaysia	Roche Elecsys hs-cTnT	Depends on an attending physicians (within 3 h)	None	Less than 14 ng/L
Kuala Lumpur Hospital	Malaysia	Roche Elecsys hs-cTnT	Depends on an attending physicians (within 3 h)	None	Less than 14 ng/L
Institute Janutng Negera	Malaysia	Roche Elecsys hs-cTnT	Depends on an attending physicians (within 3 h)	None	Less than 14 ng/L

### Intervention condition: the 0/1-h algorithm

The 0/1-h algorithm uses the blood concentrations of hs-cTnT obtained at time of ED presentation and their absolute changes within 1 h to classify patients into the rule-out, observe, or rule-in group [8]. Patients with an hs-cTnT concentration <12 ng/L at 0h and a 1hΔ <3 ng/L will be stratified into the rule-out group. In addition, a patient can be directly stratified into the rule-out group if hs-cTnT concentrations is <5 ng/L at 0h and chest pain onset was more than 3 h prior to ED presentation without the need for further serial hs-cTnT-testing. Those patients with hs-cTnT ≥52 ng/L or a 1hΔ ≥5 ng/L will be stratified into the rule-in group. The remaining patients will be placed into the observe group. Patients triaged towards rule-out may be considered for discharge from the ED after the exclusion of other life-threatening disorders causing chest pain (e.g., pulmonary embolism, aortic dissection, pneumothorax) according to the patients’ conditions and the judgment of the treating physicians. Treating physicians are free to overrule any triage recommendation of the ESC 0/1-h algorithm whenever deemed necessary. Written informed consent was obtained from all patients in this phase. Adherence of the algorithm was assessed as the percentage of patients managed without protocol violations (e.g., rule-out of NSTEMI with a single measurement [rather than 2] in early presenters, defined as patients presenting within the first 3 h after chest pain onset).

### Transition period: implementation support

To support the implementation of the 0/1-h algorithm during the transition period, we will provide written educational material and presentations at each site as well as training for clinical and laboratory staff. Educational material on the algorithm and decision thresholds will be presented at each ED handover (twice daily) during the transition period to ensure wide coverage of staff on all shift patterns. This will be reinforced by specialist chest pain nurses who will receive detailed training prior to implementation and who support ED clinicians in assessing patients with suspected ACS. Key details from the educational presentation will be formatted into a one-page reference guide that will be posted within each department and online in the hospital guidelines portal. This information will also be presented to the wider hospital teams in medical grand round presentations prior to implementation and circulated to all general practitioners. Laboratory staff will also receive training to ensure that any queries directed to the laboratory will be dealt with consistently. Finally, the research team will include senior cardiologists, emergency physicians, and cardiology nurses who are clinically active within each of the hospital clusters; education will therefore be reinforced at a local level by these clinical leaders throughout the transition period. This period will not be subject to an analysis.

### Randomization

Simple randomization will be performed. The randomization sequence of the participating clusters will be generated using computer-generated pseudo-random numbers by a statistician at the Japanese Organisation for Research and Treatment of Cancer (JORTC), who will be not otherwise involved in the study.

### The adjudicated final diagnosis

Both components of the primary outcome are adjudicated by two independent cardiologists. Interrater reliability is assessed by documenting the number of patients with mismatch in the final diagnosis of AMI by the 2 adjudicating cardiologists, which requires involvement of a third cardiologist. AMI will be defined according to the Fourth Universal Definition of Myocardial Infarction, which requires evidence of myocardial necrosis in addition to ischemia (as shown through ECG changes, positive findings of stress ECG, myocardial perfusion, coronary CTA or CAG) [25]. In brief, necrosis will be diagnosed based on an increase or decrease (20% relative increase) in the hs-cTnT concentration with at least 1 measurement above the 99th percentile of the normal reference range at a level of assay imprecision near 10%. Type I MI is characterized by atherosclerotic plaque rupture, ulceration, erosion, or dissection, with the resulting intraluminal thrombus in one or more coronary arteries leading to myocardial necrosis (any hs-cTnT concentration above 14 ng/L, with an increase and decrease in the hs-cTnT concentration where serial testing is available). Type II MI is defined as myocardial necrosis in which a condition other than coronary plaque instability contributed to an imbalance between the myocardial oxygen supply and demand (e.g., coronary artery spasm, coronary embolisms, hypertension, or hypotension). All other patients will be classified as “no AMI” in this analysis. Unstable angina will be diagnosed in patients with typical ischemic symptoms at rest or minor exercise with no evidence of acute myocardial necrosis (normal or elevated hs-cTnT levels without any relevant change during serial sampling). Vasospastic angina pectoris was diagnosed in patients with no obvious coronary stenosis, in which an ECG revealed ST elevation, or there was angiographic evidence of a coronary artery spasm that was released after the intracoronary administration of 1 mg of nitroglycerin [18, 26, 27]. Noncardiac chest pain was diagnosed based on the absence of findings from laboratory tests, ECG, and chest radiography at the 30-day follow-up.

### Adherence

The principle investigator have regular web meeting (every the other month) with local PI and their colleagues.

### Study governance

The steering committee of emergency physicians, emergency and cardiology nurses, cardiologists, implementation experts, and clinical pathologists with representation from each of the participating hospitals is providing oversight to the study. Data management is centralized at Novelle PACE Inc. (Tokyo, Japan). REDCap (Research Electronic Data Capture) will be used as electronic case report form (eCRF) to collect and manage study data. All local and server databases will be secured with password-protected access systems, access logging, and encryption. Members of the endpoint committee have access to the patient information via encrypted server access and encrypted hard drives. A clinical event adjudication committee, independent of the study management team, is providing blinded evaluations (events deidentified for treatment arm, hospital, and patient details) of all components of the primary endpoint, including index (within 12 h of initial presentation) and subsequent MI. All study-related information will, until collection, be stored securely at the study sites in areas with limited access. After collection and entry, laboratory specimens, reports, and data collection forms will be identified by a coded participant ID number to maintain participant confidentiality. Forms and any other listings that link participant ID numbers to other identifying information will be stored separately from study records with the participant ID number.

### Study funding

This is an investigator-initiated study funded by a Grant-in-Aid for Scientific Research (No. 18K09954) with additional funding through a restricted grant from Roche Diagnostics. Roche Diagnostics were approached after the study was designed, ethical approval gained, and enrollment commenced, and their contribution was not dependent on any protocol modification or direct access to the study data.

### Statistical analyses

Continuous variables will be presented as the mean (standard deviation) or median (interquartile range [IQR]), and categorical variables will be presented as numbers and percentages. The 30-day incidence of MACEs will be analyzed using generalized linear mixed models after adjusting for background information and seasonal effects. The clustering effect by hospital will be considered in this analysis as a random effect. The binomial distribution and identity link will be used to directly estimate the absolute differences in MACE incidence between 0/1-h algorithm care and usual care patients. The main model will include conditions (control or intervention) and steps (time periods) as categorical variables and hospitals as clusters. Differences in MACEs with a 1-sided 95% CI will be estimated in order to evaluate non-inferiority. The non-inferiority margin is preset at 1.5%. The sensitivity and negative predictive values (NPVs) for MACEs in the rule-out group and the specificity and positive predictive values (PPVs) for MACEs in the rule-in group will be calculated. Multiple imputations will be used to handle missing data. The statistical analyses will be performed using the SAS software program, version 9.4 (SAS Institute, Cary, NC, USA), and R version 4.0.3 (R Foundation for Statistical Computing, Vienna, Austria).

### Auditing

This study does not have a dedicated research audit department. There is no independent Trial Auditing Committee. Instead, the research team reviews recruitment rates every 2 weeks and meets on every 2-month basis to discuss key milestones as well as the conduct of the research, ensuring the integrity of the protocol and conduct of the study.

### Protocol amendment

Any significant amendments to the study protocol will be provided to and approved by the Ethical Review Board of Juntendo University Nerima Hospital before implementation. If approved, these changes would have to be reported in the trial register.

### Ancillary and post-trial care

There is no ancillary post-trial care or compensation. Patients are enrolled in routine therapy during the study duration.

### Dissemination policy

The trial results will be published in a peer-reviewed journal. The results will also be presented at scientific conferences. A popular science summary of the results will be posted online for laymen and study participants. An anonymized data set as well as statistical code used to analyze the data will be published in a data repository on open science framework.

### Patient and public involvement

A patient review panel was consulted throughout the trial program and provided input on the educational advice provided to clinicians after the introduction of the new pathway. Patients were not involved in the conception or design of the trial.

### Cost-effectiveness analyses

These analyses will be performed in six hospitals in Japan, Taiwan, and Thailand, only, as the remaining centers participating in this study lack a system for conducting cost analyses.

#### Japan

The diagnosis procedure combination (DPC; flat-fee payment system) is the bundled payment system of medical fees for acute inpatient medical care in Japan [28].

The bundled payment for each hospitalization will be calculated according to the codes in the International Classification of Diseases 10th revision (ICD-10) and the coefficient for each facility. The DPC database contains six categories of diagnoses, each with a limited number of recordable diseases. One diagnosis each is coded for the “main diagnosis,” “admission-precipitating diagnosis,” “most resource-consuming diagnosis,” and “second-most resource-consuming diagnosis.” A maximum of 4 diagnoses (10 diagnoses from 2016) each can be coded for “comorbidities present at time of admission” and “conditions arising after admission.” All procedures performed during hospitalization and outpatient management are recorded according to the Japanese fee schedule for reimbursement. The DPC component corresponds to the so-called “hospital fee,” including the basic hospital charge, pharmaceuticals, injections, laboratory examinations, and other related expenses, and is paid on a per-day payment scheme. The fee-for-service (FFS) component corresponds to the charges for surgical procedures, angiography, and other related expenses. The revenue equals the sum of the DPC and FFS components. “Material costs” include the costs of pharmaceuticals and medical materials. The total hospitalization costs are calculated as the sum of bundled payment and FFS, without the food fee [29]. The data will be anonymized and not include any information that might be used to identify individuals or hospitals. Each patient will have an identifier specific to the hospitals, with all patients regarded as a single individual, regardless of having both inpatient and outpatient data. The DPC data used for payments include patient demographics and select clinical information, admission and discharge statuses, diagnoses, surgeries and procedures performed, medications, and special reimbursements for specific conditions. In this study, the DPC data will be evaluated from admission to the 30-day follow-up period. The data will be excluded if patients receive care from a non-cardiology department.

#### Taiwan

The DPC is the payment system of medical fees for acute patient medical care in Taipei City Hospital. The payment will be calculated according to the medical cost requested from the National Health Insurance program. The database contains cardiologic-related diagnoses. Each patient is coded for the “main diagnosis,” “admission-precipitating diagnosis”, “every comorbidity at every admission,” and “second-most resource-consuming diagnosis.” All procedures performed are recorded according to the Taiwanese fee schedule for reimbursement. The total cost component corresponds to the so-called “hospital fee,” including the basic hospital charge, pharmaceuticals, injections, laboratory examinations, surgical procedures angiography, and other related expenses, and is paid on a per-day payment scheme. The total costs are calculated as the sum of all medical costs, without the food fee, which is recorded in the hospital computer system.

#### Thailand

The cost for each participant will be identified from the hospital electronic database. The total cost will be disaggregated into individual cost items, including drugs, laboratory fees, and service costs, such as interventions, operations, and room costs, incurred at Maharaj Nakorn Chiang Mai Hospital, Faculty of Medicine, Chiang Mai University.

## Discussion

### Concerns about the 0/1-h algorithm

Cardiac troponin is the preferred biomarker of myocardial injury owing to its high specificity and sensitivity. In patients with suspected NSTE-ACS, serial sampling should be performed at presentation and after 3–6 h to demonstrate the existence of a rise and/or fall in troponin (or even earlier when high-sensitivity assays are used), as recommended since the third UDMI in 2012. The 0/1-h algorithm is recommended by the ESC guideline, based on its high safety and efficacy observed in multiple observational studies [14]. However, the clinical implementation of the 0/1-h algorithm in Europe and recently Northern America has not been without controversy due to the fact that the majority of the findings related to the 0/1-h algorithm were derived from highly controlled settings of observational, diagnostic studies [30, 31]. Similarly, the 0/1-h algorithm was derived in Central European patients. Shiozaki el al. reported Japan-Taiwan data [18, 26, 32]; however, particularly patients from Asian and African countries are largely underrepresented in the existing validation cohorts. As a result, the 0/1-h algorithm has not been introduced at some institutions in regular clinical practice.

### Characteristic of Asia

Compared with western countries, some countries in Asia showed much lower rates of coronary angiography and revascularization for patient with ACS, and also some of the highest post-discharge mortality rates [12, 22, 33–36]. This discrepancy highlights that quality of care is not only a matter of availability of resources but also depends on knowledge of guidelines and application of its recommendations, organization of care, including effective referral systems from secondary centers to tertiary centers with full coronary revascularization facilities, and issues related to social security coverage and/or need for reimbursement for procedures. The 0-h/1-h algorithm could aid the rapid transfer of high-risk patients to specialist cardiac facilities. These challenges can be best met by establishing cardiac networks and individual hospital models/clinical pathways taking into account local risk factors (including socioeconomic status), affordability and availability of pharmacotherapies/invasive facilities, and the nature of local healthcare systems.

### Study purpose

There are three complete randomized controlled trials that have been performed in this area. Two are from UK [24, 37], and the other is from Australia [10]. To expand the application of the 0/1-h algorithm in Asian countries, we intended to conduct a prospective, stepped wedge, cluster-randomized controlled approach, which will help evaluate the overall safety and efficacy of this novel approach. Asian countries are highly relevant as they represent a very large population with heterogenous medical settings. In general, when assessing novel diagnostic approaches with the potential to improve efficacy, clinicians always require evidence that safety of such a decision rule is non-inferior to standard of care. We also intend to compare the ED dwelling time as well as medical costs among Japan, Thailand, and Taiwan as a secondary endpoint. We hypothesize that the implementation of the 0/1-h algorithm has the potential to achieve an improved cost performance by the shortened ED dwelling time as well as the avoidance of unnecessary admissions or costly downstream cardiac examinations.

### Limitations

Our study is associated with several limitations. First, while the study is powered to document non-inferiority in safety of the 0/1-h algorithm compared with standard care, the targeted sample size does not allow to test for superiority in efficacy. Nevertheless, given the real-world design of this study with broad inclusion criteria, observations made within this study will provide important insights into the clinical utility of the 0/1-h algorithm in all-comers. Second, as the primary outcome, we assess the incidence of MACE within 30 days after index presentation in outpatients, which is the most relevant and best accepted measure to quantify safety of any triage algorithm in suspected ACS. However, we cannot comment on outcomes that occur beyond 30 days. Third, only Japan has a national registry named DPC to evaluate a whole medical cost related with the medical service in a following period. Other countries do not have. Therefore, in both Taiwan and Thailand, we will evaluate it in each hospital, individually. In the remaining centers, no data on cost-efficacy will be collected, which could cause some selection bias. Fourth, we cannot comment on the utility of the 0/1-h algorithm in patients with terminal kidney failure on chronic dialysis, because these patients were excluded from the initial studies deriving and validating this algorithm. Thus, patients on chronic dialysis will be excluded also in this study. Fifth, some selection bias may occur due to the need for consent in the intervention arm. Sixth, Juntendo University Nerima Hospital Juntendo University Urayasu hospital, and Tapei city hospital had a history to implement the 0/1-h algorithm. But during the usual care period, second measurement of troponin at 1-h interval will be prohibited.

## Conclusion

Asian countries represent the largest population in the world, but the region comprises many island nations, with no unified trends. As a result, there are regional differences in the degree of congestion in the ED. However, the degree of congestion is associated with a worsening of the patient prognosis, especially regarding patients with chest pain disease. The introduction of the 0/1-h algorithm is therefore expected to maintain high safety while reducing medical costs by shortening the medical treatment time, utilizing efficient patient stratification based on quantitative evaluations, and reducing the performance of excessively invasive medical treatments and unnecessary hospitalization. The national medical systems of these countries differ markedly, as do approaches to financing medical care.

### Trial status

The study is currently recruiting patients. As of Sep 30th, 2022, 2124 patients had been included. The first patient was included on July 1st, 2020, and inclusion is expected to be completed on October 14th, 2023.

## Data Availability

The final dataset will not be accessible for the research group of the DROP-Asian ACS study (the authors of this protocol). The datasets generated and/or analyzed during the current study are not publicly available, owing to protection of personal data.
