# Correction: An international, stepped wedge, cluster-randomized trial investigating the 0/1-h algorithm in suspected acute coronary syndrome in Asia: the rational of the DROP-Asian ACS study

**DOI:** 10.1186/s13063-023-07342-9

**Published:** 2023-05-17

**Authors:** Kenji Inoue, Jack Tan Wei Chieh, Lim Chiw Yeh, Shuo-Ju Chiang, Arintaya Phrommintikul, Pannipa Suwanasom, Sazzli Kasim, Bakhtiar Ahmad, Alzamani Mohammad Idrose, Farina Mohd Salleh, Shunsuke Oyamada, Yohei Hirano, Shohei Ouchi, Moriyuki Terakura, Naoyuki Yokoyama, Ken Kozuma, Mamoru Nanasato, Ryosuke Higuchi, Kazuhiko Yumoto, Tomoyuki Fukuzawa, Issei Shimada, Evangelos Giannitsis, Raphael Twerenbold, Tohru Minamino

**Affiliations:** 1 Department of Cardiovascular Biology and Medicine, Juntendo University Nerima Hospital, Tokyo, Japan; 2 Department of Cardiology, National Heart Centre Singapore and Sengkang General Hospital, Singapore, Singapore; 3 Division of Cardiology, Department of Internal Medicine, Taipei City Hospital Yangming Branch, Taipei, Taiwan; 4 Division of Cardiology, Department of Internal Medicine, Faculty of Medicine, Chiang Mai University, Chiangmai, Thailand; 5 Division of Cardiology, Hospital Al-Sultan Abdullah, University Teknologi MARA, Kuala Lumpur, Malaysia; 6 Division of Emergency, Kuala Lumpur Hospital, Kuala Lumpur, Malaysia; 7 Division of Emergency, Institut Jantung Negara, Kuala Lumpur, Malaysia; 8 Departments of Biostatistics, JORTC Data Center, Tokyo, Japan; 9 Department of Emergency and Critical Care Medicine, Juntendo University Urayasu Hospital, Chiba, Japan; 10 Department of Cardiovascular Biology and Medicine, Juntendo Urayasu Hospital, Chiba, Japan; 11 Department of Emergency, Teikyo University School of Medicine, Tokyo, Japan; 12 Department of Cardiology, Teikyo University School of Medicine, Tokyo, Japan; 13 Department of Cardiology, Sakakibara Heart Institute, Tokyo, Japan; 14 Department of Cardiology, Yokohama Rosai Hospital, Kanagawa, Japan; 15 Shimada General Hospital, Chiba, Japan; 16 Department of Cardiology, University Hospital Heidelberg, Heidelberg, Germany; 17 Department of Cardiology and University Center of Cardiovascular Science, University Heart and Vascular Center Hamburg, Hamburg, Germany; 18 Department of Cardiovascular Biology and Medicine, Juntendo University Graduate School of Medicine, Tokyo, Japan

**Correction: BMC Trials (2022) 23:986**

**https://doi.org/10.1186/s13063-022-06907-4**

Following publication of the original article [1], we have been informed that the below phrase in the Methods section of the Abstract needs to be modified:

Originally published:

Consecutive patients presenting with suspected acute coronary syndrome between July 2022 and Apr 2024 were included.

Corrected phrase (2022 becomes 2021):

Consecutive patients presenting with suspected acute coronary syndrome between July 2021 and Apr 2024 were included.

The original article has been corrected.

## References

[1] Inoue K (2022). BMC Trials..

